# Cirrhosis in Transition: Emerging Concern in a Post-Pandemic World

**DOI:** 10.5152/tjg.2024.0201251

**Published:** 2025-12-30

**Authors:** Dilara Turan Gökçe, Meral Akdoğan

**Affiliations:** 1 Department of Gastroenterology, Sincan Education and Research Hospital, Ankara, Türkiye; 2 Department of Gastroenterology, Ankara Bilkent City Hospital, Ankara, Türkiye

In recent years, the etiology of chronic liver diseases has been undergoing significant changes due to advancements in treatment options, the COVID-19 pandemic, and shifting lifestyles. We read with great interest the article by Üçbilek et al., titled “Trends in the Etiology of Cirrhosis,” published in your esteemed journal.^1^ This study evaluated cirrhosis etiologies before and after 2010, including data up to 2020. It demonstrated an increase in the prevalence of cirrhosis associated with metabolic dysfunction-associated liver disease (MASLD), while the proportion of cirrhosis due to viral hepatitis has declined. In this manuscript, we aim to shed light on new research by assessing recent data and sharing our own experiences in this field.

Firstly, as highlighted in your study, it is undeniable that the prevalence of MASLD-related cirrhosis is steadily increasing globally, paralleling rising obesity and diabetes rates. In the United States, liver transplants due to metabolic dysfunction-associated steatohepatitis (MASH)-related cirrhosis rose from 2.5% in 2004 to 20.4% in 2019.^2^ Similar trends are observed worldwide, and this increase is expected to persist in Turkey, one of the European countries with the highest obesity prevalence. The long-term impact of medical, endoscopic, and surgical treatments targeting obesity on this trend will only become evident over time.

The global incidence of alcohol-related liver disease (ALD) has also increased in recent years. A study focusing on U.S. data predicts that if current trends continue unchecked and new policies are not implemented, the age-standardized incidence of alcohol-related decompensated cirrhosis could rise by 77%, increasing from 9.9 cases per 100,000 patient-years in 2019 to 17.5 cases per 100,000 patient-years by 2040.^3^ While insufficient data exist for Turkiye, clinical observations indicate an increasing frequency of ALD among young individuals and women. For instance, at our center, the proportion of liver transplants due to ALD-related cirrhosis rose from 3% between 2001–2018 to 6.6% between 2019–2024. Increased alcohol consumption is expected to further elevate this proportion in the coming years.

A study investigating the etiology of hepatocellular carcinoma (HCC) in Turkiye, involving data from 1,802 patients across 14 centers between 2001 and 2020, revealed that hepatitis B virus (HBV) remains the most common etiological factor (54%).^4^ While viral hepatitis accounted for 80.5% of cases before 2016, this proportion significantly decreased to 72.5% after 2016. Biennial analyses within the same study indicate a declining share of viral hepatitis in the etiology of HCC, alongside an increasing frequency of HCC associated with steatotic liver disease.^4^ More extensive and updated data, particularly incorporating post-COVID-19 data, are needed in Turkiye to address these trends comprehensively.

While the present study reflects data up to 2020, clinical observations and post-2020 data indicate a rising prevalence of autoimmune and autoinflammatory diseases. A significant recent study from the United Kingdom examining changes in the prevalence of primary sclerosing cholangitis (PSC) and PSC-associated inflammatory bowel disease (PSC-IBD) reported an increase from 5.0 per 100,000 population in 2015 to 7.6 per 100,000 in 2020.^5^ Including patients who developed inflammatory bowel disease (IBD) after PSC diagnosis, the prevalence rose from 5.7 in 2015 to 8.6 in 2020. The annual average percentage change (AAPC) in PSC-IBD prevalence between 2015 and 2020 was 8.8%, higher than the rate for IBD alone (7.0%). The prevalence of PSC-IBD is projected to reach 11.7 per 100,000 population by 2027 (or 13.3 per 100,000 including patients who develop IBD after PSC diagnosis).^5^ These findings suggest that the growth rate of PSC prevalence exceeds that of IBD. At our center, PSC cases among liver transplant patients rose from 1.8% between 2003–2018 to 11% between 2019–2024. Similarly, especially following the COVID-19 pandemic, we have observed an increase in resistant autoimmune hepatitis cases and their growing role in the etiology of cirrhosis. Enhancing global and national data on this subject is crucial for guiding effective disease management strategies.

The rates of cirrhosis, HCC, and liver transplants due to HBV and hepatitis C virus (HCV) infections have been declining worldwide. Similar reductions are expected in Turkiye, thanks to the nationwide HBV vaccination program initiated in 1998 and the availability of direct-acting antivirals (DAAs) for HCV. In the study by Üçbilek et al., the proportion of HCV in the etiology of cirrhosis decreased from 12% to 11% over 10-year periods.^1^ However, the accessibility and impact of DAAs after 2015 would likely result in a more pronounced decline in HCV-related cirrhosis. The broad 10-year interval of the analysis may have obscured this effect. Unfortunately, the COVID-19 pandemic partially slowed the impact of global treatment and vaccination programs. During the pandemic, the initiation of new antiviral treatments for HCV declined significantly, with a 51% reduction in Europe and a 54% decrease outside Europe.^6^ This has disrupted the global HCV eradication program targeted for 2030.

In conclusion, MASLD-related cirrhosis is likely to remain a significant concern for the foreseeable future. Similarly, cirrhosis associated with autoimmune hepatitis and PSC, conditions with still unclear etiologies, is expected to gain increasing attention. Moreover, the insufficient societal efforts to address alcohol use disorder will further prolong the challenges of combating alcohol-related cirrhosis. Advancing the understanding of these diseases, improving management strategies, and prioritizing preventive measures should be recognized as critical global health priorities.

## Data Availability

The data that support the findings of this study are available on request from the corresponding author.
